# Posthemorrhagic ventricular dilatation late intervention threshold and associated brain injury

**DOI:** 10.1371/journal.pone.0276446

**Published:** 2022-10-27

**Authors:** Eva Valverde, Marta Ybarra, Andrea V. Benito, María Carmen Bravo, Adelina Pellicer

**Affiliations:** 1 Department of Neonatology, La Paz University Hospital, Madrid, Spain; 2 NeNe Foundation, Madrid, Spain; 3 Hospital La Paz Institute for Health Research-IdiPAZ, Madrid, Spain; Western University, CANADA

## Abstract

**Objective:**

To systematically assess white matter injury (WMI) in preterm infants with posthemorrhagic ventricular dilatation (PHVD) using a high-threshold intervention strategy.

**Study design:**

This retrospective analysis included 85 preterm infants (≤34 weeks of gestation) with grade 2–3 germinal matrix-intraventricular hemorrhage. Cranial ultrasound (cUS) scans were assessed for WMI and ventricular width and shape. Forty-eight infants developed PHVD, 21 of whom (intervention group) underwent cerebrospinal fluid drainage according to a predefined threshold (ventricular index ≥p97+4 mm or anterior horn width >10 mm, and the presence of frontal horn ballooning). The other 27 infants underwent a conservative approach (non-intervention group). The two PHVD groups were compared regarding ventricular width at two stages: the worst cUS for the non-intervention group (scans showing the largest ventricular measurements) versus pre-intervention cUS in the intervention group, and at term equivalent age. WMI was classified as normal/mild, moderate and severe.

**Results:**

The intervention group showed significantly larger ventricular index, anterior horn width and thalamo-occipital diameter than the non-intervention group at the two timepoints. Moderate and severe WMI were more frequent in the infants with PHVD (p<0.001), regardless of management (intervention or conservative management). There was a linear relationship between the severity of PHVD and WMI (p<0.001).

**Conclusions:**

Preterm infants with PHVD who undergo a high-threshold intervention strategy associate an increased risk of WMI.

## Introduction

The immature preterm brain is at high risk of developing germinal matrix-intraventricular hemorrhage (GMH-IVH) and white matter injury (WMI), conditions that are associated with abnormal brain growth and neurodevelopmental impairment [1]. The prevalence of GMH-IVH among preterm infants (<32 weeks gestation) reaches 25%, and a relevant subset of these infants will experience severe bleeding (7%–18%) [2]. GMH-IVH entails complications such as periventricular hemorrhagic infarction (PVHI), posthemorrhagic ventricular dilatation (PHVD) [2–4], and the need for intervention to remove cerebrospinal fluid, either by using a ventricular reservoir or by ventricular shunt insertion (VSI) [4, 5]. Severe GMH-IVH has been associated with motor and neurocognitive deficits, particularly in infants who develop PHVD requiring VSI [2–4, 6–12]. Brain growth is also significantly impaired in PHVD [13, 14], and the neuropathological consequences of GMH-IVH on white and gray matter and the cerebellum have been established [2, 13–15]. The pathophysiology appears to be related to increased pressure, causing vasospasm, neuroinflammation and secondary periventricular WMI [16–21]. Although the objective of cerebrospinal fluid removal in infants with significant ventricular dilatation is to prevent further damage [22–25], there is still no consensus regarding the timing and type of intervention [4] or on the actual benefit of secondary brain injury prevention. The impact of moderate-severe GMH-IVH on neurodevelopmental outcomes is difficult to characterize, given the heterogeneity of studies on the subject, differences in PHVD management, and the lack of studies documenting the extent of associated parenchymal brain injury with a determinant role on outcomes [26, 27].

The purpose of this retrospective analysis of a cohort of preterm infants below 34 weeks of gestation was to evaluate our routine approach and intervention strategy in PHVD, based on a systematic assessment of the ventricular width and shape, with a high-threshold intervention (as defined by De Vries et al. [5]), with a particular focus on the associated WMI.

## Methods

This review includes all data sets of preterm infants with less than 34 weeks gestation admitted to our level 3 neonatal intensive care unit (NICU), who were diagnosed with grade 2–3 GMH-IVH or PHVD between January 2014 and December 2018. Only inborn (those delivered at the NICU) and outborn (those delivered at another healthcare facility and then transferred to the NICU) infants who had a cranial ultrasound (cUS) study during the first two weeks of birth and subsequent serial studies and, who survived beyond day 10 of life were included in the final analysis. All clinical data were extracted from the medical records. The need for informed consent was waived by the Medical Research Ethics Committee of La Paz University Hospital (Madrid) who approved the study.

### Cranial ultrasound and PHVD intervention protocol

The routine cUS protocol for high-risk preterm infants at our institution includes early (at least two scans during the first week), serial (every 1 or 2 weeks, depending on cUS findings), and term equivalent age (TEA) exams. The images are stored in digital format, allowing for a complete reassessment of brain parenchyma and measurements of several indexes of interest at any time. For the present study, all scans were independently reviewed by two experienced examiners (MY, EV) according to the predefined diagnoses (below). Disagreements were resolved by consensus meetings.

Using parasagittal views, GMH-IVH was classified as grade 2 if the intraventricular bleeding occupied less than 50% of the ventricular width and as grade 3 if more than 50% of the ventricular width was filled by blood and there was acute dilatation of the lateral ventricles [2].

The ventricular width was defined according to three parameters: 1) the ventricular index (VI), defined as the distance between the cerebral falx and the lateral wall of the anterior horn in the coronal plane; 2) the anterior horn width (AHW), defined as the diagonal width of the anterior horn measured at its widest point in the coronal plane; and 3) the thalamo-occipital distance (TOD), defined as the distance between the outermost point of the thalamus at its junction with the choroid plexus and the outermost part of the occipital horn in the parasagittal plane [28]. PHVD was established whenever VI was more than 2 standard deviations (SD) for postmenstrual age [29] or AHW was >6 mm.

The intervention criteria for PHVD was based on ventricular width (VI ≥p97+4 mm or AHW >10 mm) and the presence of frontal horn ballooning (defined as a change in ventricular shape consisting of a rounding of the frontal horn). Cerebrospinal fluid was removed either by a ventricular reservoir or VSI, depending on whether the patient weighed less than or more than 1500 g. A second intervention was performed to switch from a ventricular reservoir to VSI in infants with persistent ventricular dilatation despite regular cerebrospinal fluid removal. No lumbar punctures were performed, because they are not standard practice in our unit.

WMI was classified according to a modified version of the criteria of Agut et al. [30]. Moderate WMI was defined as periventricular hyperechodensities with heterogeneous appearance, localized cyst formation and/or one or two ultrasound signs of white matter loss at TEA. Severe WMI was defined as periventricular hyperechodensities that progressed into extensive cystic lesions or were followed by more than two signs of white matter loss at TEA. Ultrasound indicators of white matter loss at TEA were established using only the ventricular width criteria: VI >13 mm, AHW >3 mm, or TOD >24 mm. Lastly, white matter was classified as normal if there were no abnormalities (no WMI) or transient periventricular echodensities (mild WMI).

The three ventricular width estimators (VI, AHW, and TOD) employed for the analysis corresponded to the scans obtained at two study timepoints: 1) the last cUS performed prior to the intervention in the patients who underwent cerebrospinal fluid removal (the intervention group) and the scan where these estimators reached their maximum value (worst cUS) in the patients with conservative approach (no cerebrospinal fluid removal), hereinafter referred to as the non-intervention group and 2) the exam conducted at TEA in the intervention and non-intervention groups.

### Statistical analysis

Data were analyzed using SPSS for Windows version 23. The quantitative data are expressed as means and standard deviation or medians and interquartile range (IQR), while the qualitative variables were expressed as absolute values and percentages. When considered, box plots were employed to display the data distribution and dispersion. The Mann-Whitney U or Kruskal-Wallis tests were used to compare the nonparametric variables, and matched samples were compared using Wilcoxon’s signed rank test. Fisher’s exact and chi-squared tests were employed to compare the categorical variables among the groups. The linear-by-linear test was employed to examine the relationship between two categorical variables. We constructed a multivariate analysis to assess the association between perinatal (multiple pregnancy, restricted intrauterine growth, delivery route, antenatal steroid use, magnesium sulfate administration and antibiotic prescription) and neonatal (sex, gestational age, birth weight, need for advanced resuscitation, cardiovascular support within the first 72 h after birth, patent ductus arteriosus requiring treatment, presence of necrotizing enterocolitis, nosocomial sepsis, use of mechanical ventilation, postnatal steroids, and GMH-IVH parameters) factors as independent variables and the development of PHVD as the dependent variable. Statistical significance was set at p<0.05.

## Results

**Fig 1:**
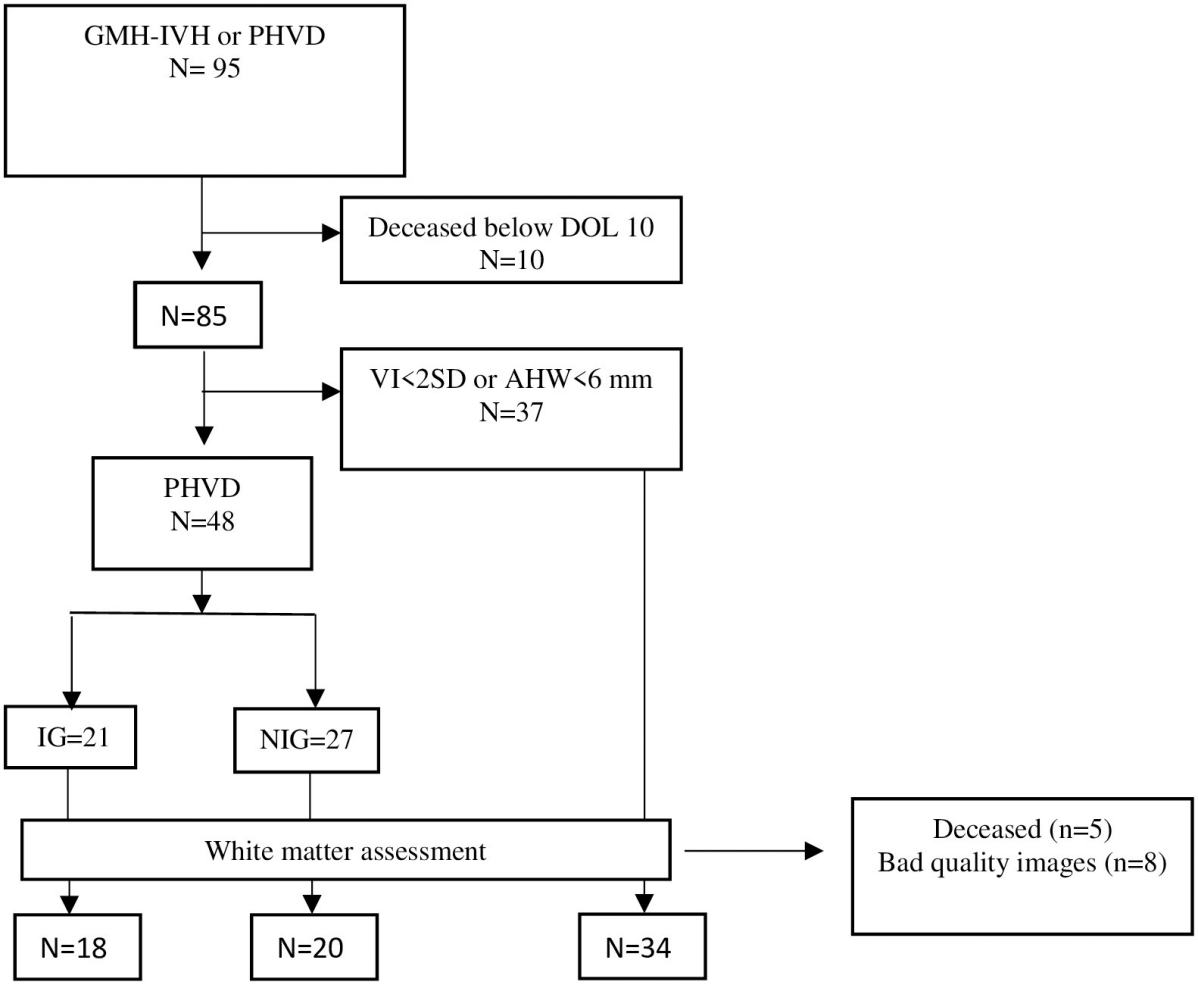
Flow chart of the study population. Flowchart of eligible infants (n = 95) and excluded infants (n = 10 who died before DOL 10 and n = 37 who had no PHVD threshold criteria). Forty-eight infants with PHVD were included in the final analysis. Abbreviations: GMH-IVH, germinal matrix hemorrhage-intraventricular hemorrhage; DOL, day of life; PHVD, post-hemorrhagic ventricular dilatation; VI, ventricular index; AHW, anterior horn width. IG, intervention group; NG, non-intervention group.

During the study period, 95 infants with less than 34 completed weeks of gestation were admitted with the diagnosis of grade 2–3 GMH-IVH or PHVD, 85 of whom were considered eligible for analysis. Forty-eight of the candidates fulfilled the diagnostic criteria of PHVD, while for the remaining 37, the ventricular width stabilized below the established threshold. The patients’ chart flow and exclusion criteria are shown in Fig 1.

The study population’s relevant clinical data are shown in Table 1.

**Table 1:** Perinatal and neonatal clinical data by study group.

	No PHVD	PHVD present			P	
	Total N = 37	Total N = 48	Intervention N = 21	No intervention N = 27	PHVD absent vs. present	PHVD intervention vs. no intervention
**Mean gestational age, weeks (SD)**	26.6 (2.6)	26.9 (2.2)	28 (2.4)	25.9 (1.5)	-	0.0001
**Mean body weight, g (SD)**	951 (341)	1036 (308)	1160 (310)	938 (275)	-	<0.01
**Male, n (%)**	23 (62)	32 (67)	14 (67)	18 (67)	-	-
**Multiple birth, n (%)**	12 (32)	15 (31)	10 (48)	5 (18)	-	**-**
**Intrauterine growth restriction, n (%)**	6 (16)	5 (10)	3 (14)	2 (7)	-	-
**Antenatal steroids, n (%)**	27 (75)	22 (46)	10 (48)	12 (44)	0.008	-
**Antenatal magnesium sulfate, n (%)**	24 (67)	16 (33)	5 (24)	11 (41)	0.004	-
**Caesarean section, n (%)**	25 (69)	36 (77)	18 (90)	18 (67)	-	**-**
**Chorioamnionitis, n (%)**	19 (53)	19 (39)	5 (24)	14 (52)	-	-
**PDA surgical treatment, n (%)**	9 (24)	8 (16)	2 (9.5)	6 (22)	-	-
**Necrotizing enterocolitis surgery, n (%)**	5 (13)	5 (19)	2 (9.5)	3 (11)	-	-
**Nosocomial sepsis, n (%)**	23 (62)	36 (75)	13 (62)	23 (85)	-	-
**Bronchopulmonary dysplasia, n (%)**	19 (51)	16 (33)	7 (33)	9 (33)	-	-
**GMH-IVH grade 2, n (%)**	30 (81)	5 (10)	1 (4.8)	4 (15)	0.0001	-
**GMH-IVH grade 3, n (%)**	7 (19)	43 (89)	20 (95)	23 (85)	0.0001	-
PVHI, n (%)	5 (13)	19 (39)	9 (43)	10 (37)	0.004	-
**Exitus between DOL 10 and TEA**	5	6	1	5	-	-

Quantitative variables are expressed as mean (SD) and categorical values are expressed as n (%). Statistical significance was set at p<0.05.

Abbreviations: PDA, patent ductus arteriosus; PHVD, posthemorrhagic ventricular dilatation; GMH-IVH, germinal matrix-intraventricular hemorrhage; PVHI, periventricular hemorrhagic infarction; DOL, day of life; TEA, term equivalent age.

The multivariate analysis identified grade 3 GMH-IVH as the only independent risk factor associated with the development of PHVD, after adjusting by gestational age. Considering only the inborn patients (i.e., those who systematically underwent early and serial cUS scans), 43% of those with grade 2–3 GMH-IVH developed PHVD, 92% of them after severe bleeding (grade 3 GMH-IVH).

### PHVD and related interventions

Of the 48 infants with PHVD, 21 underwent cerebrospinal fluid removal (ventricular reservoir, n = 18; VSI, n = 3) at a median (IQR) age of 17 (12–41) and 31 (19–41) days, for the ventricular reservoir and VSI, respectively. A second intervention to switch from ventricular reservoir to VSI was scheduled for 15 of the 18 infants with a reservoir at a median (IQR) age of 60 (34–78) days. The complications were only related to VSI (infection, n = 2; need for replacement, n = 7).

**Fig 2:**
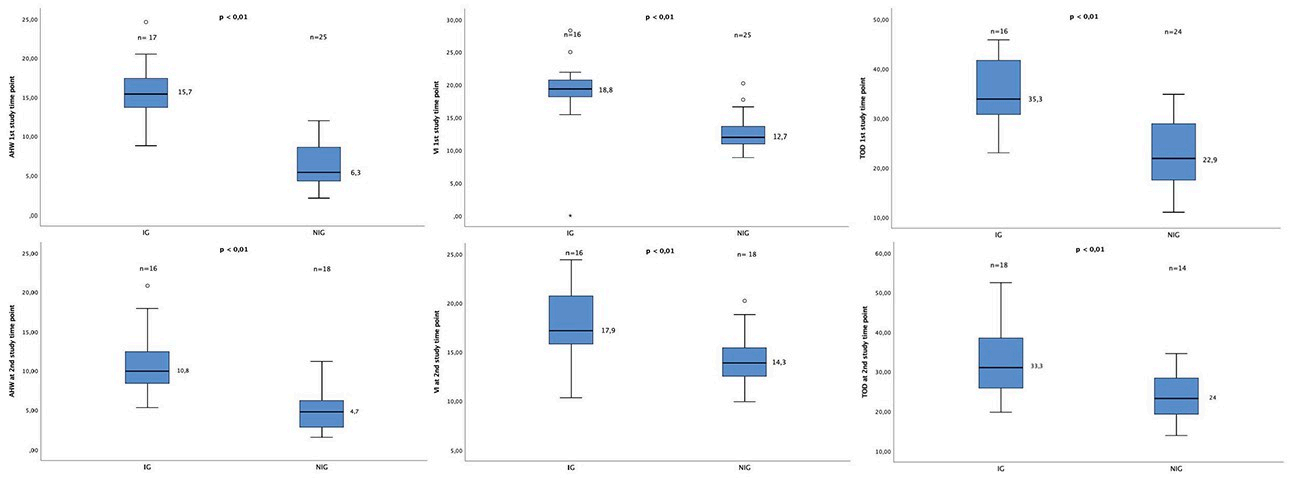
Ventricular width by study group. Ventricular measurements (AHW, IV and TOD) at the first study timepoint prior to intervention (IG) compared with the worst cUS (NG) in the upper panel, and at the second study timepoint (TEA) in lower panel. Ventricular size was significantly larger in the IG than in the NG prior to intervention and at TEA. Ventricular values are presented as median ± IQR (mm). Abbreviations: AHW, anterior horn width; IV, ventricular index; TOD, thalamo-occipital distance; IG, intervention group; NG, non-intervention group; cUS, cranial ultrasound; TEA, term equivalent age; IQR, interquantile range.

The results of the ventricular width comparisons by study group are shown in Fig 2. The intervention group had a significantly larger ventricular size than the non-intervention group prior to intervention: VI 18.8±5.8 vs. 12.7±2.8 mm (p<0.001), AHW 15.7±3.7 vs. 6.3±2.9 (p<0.001),TOD 35.3±7 vs. 22.9±6.8 (p<0.001); and at TEA: VI 18.4±3.3 vs. 14.2±2.8 (p<0.01), AHW 10.9±4.3 vs. 4.6±2,4 (p<0.01) and TOD 33.4±9.7 vs. 22.2±8.4 (p = 0.01).

### White matter assessment

Thirteen infants were excluded from the study for a white matter appraisal due to early death (n = 5) or poor scan quality for assessing brain parenchyma (n = 8), resulting in 72 infants who were assessed and classified for white matter abnormalities as normal-mild (n = 29), moderate (n = 28), or severe (n = 15). Eight infants (11%) developed extensive cystic leukomalacia. Among the infants who survived to TEA, 23 (32%) showed signs of white matter loss at TEA (ex-vacuo ventriculomegaly).

WMI was significantly more prevalent among the infants with GMH-IVH who developed PHVD than in those who did not (p<0.001). Considering the subset of infants who developed PHVD, those undergoing neurosurgical procedures (intervention group) showed higher rates of severe WMI than those who did not (non-intervention group) (p<0.001). There was a linear relationship between the PHVD severity and the degree of white matter injury (p<0.001). Table 2 shows the white matter abnormalities and group comparisons.

**Table 2:** White matter abnormalities by study group.

WMI	Absent/mild	Moderate	Severe	Total	P
**PHVD absent n(%)**	19 (56)	12 (35)	3 (9)	34	<0.001
**PHVD present n(%)**	5 (13)	20 (53)	13 (34)	38	
**PHVD-IG n(%)**	0 (0)	8 (44)	10 (56)	18	<0.001
**PHVD-NG n(%)**	5 (25)	12 (60)	3 (15)	20	

Linear relationship between severity of the PHVD and the degree of white matter injury using chi-squared linear tendency (p<0.001).

Abbreviations: WMI, white matter injury; PHVD, posthemorrhagic ventricular dilatation; IG, intervention group; NG, non-intervention group.

## Discussion

This retrospective analysis of a cohort of infants with less than 34 weeks of gestation who experienced grade 2–3 GMH-IVH showed an association between PHVD and the degree of ventricular dilatation and WMI co-occurrence and severity. The multivariate analysis concluded that grade 3 GMH-IVH was the only independent factor related to the development of PHVD, an association that has been scarcely reported [3]. GMH-IVH entails complications such as PVHI and PHVD. The progression of PHVD ranges from spontaneous arrest to progressive ventricular dilatation [3]. In our study, 43% of the infants with grade 2–3 GMH-IVH developed PHVD, most of them (92%) after severe bleeding (grade 3 GMH-IVH), which agrees with previous reports [2, 3, 5, 31]. It is difficult to know a priori the progression of the ventricular dilatation; there is therefore no consensus regarding the type and timing of intervention. If we manage to better define early specific ultrasound parameters for infants with intraventricular hemorrhage and ventricular dilatation that enables us to predict the progression more accurately, with special focus to the brain parenchymal abnormalities associated with PHVD, we will have more information for making decisions as to the timing of intervention.

Our approach was to reassess the WMI at TEA [30], observing a high prevalence of white matter loss, defined as the presence of ventricular enlargement, a feature more frequent in the infants who developed PHVD than in those who did not. White matter loss was particularly frequent and more severe in the infants who met the criteria for neurosurgical intervention. In fact, 56% of the infants with uncomplicated bleeding who did not develop PHVD had normal/mild WMI compared to 13% of the infants with PHVD (Table 2). While 56% of the infants with more severe ventricular dilatation requiring intervention showed severe WMI, only 15% of the infants with PHVD who did not meet the criteria for intervention (according to our protocol) had that diagnosis. In addition to PVHI and cystic periventricular leukomalacia [32, 33], secondary white and gray matter injury and reduced brain volumes have been documented in infants with PHVD [13, 34, 35]. Focal (non-cystic) and diffuse WMI might be underestimated by cUS and is examiner dependent [36, 37]. High-resolution ultrasound equipment, early and serial cUS, and new classifications for WMI at TEA that include the concept of white matter loss are therefore critical [30, 36–40]. In this study, two experienced examiners evaluated all the available cUS scans, taking into account the progression profile of the white matter echogenicity and the presence of cysts, as well as signs of white matter loss (ex-vacuo ventriculomegaly) in the cUS conducted at TEA, which have shown a good correlation with MRI findings [41, 42].

The ventricular width remained larger in the infants in the intervention group than in the non-intervention group at TEA, regardless of intervention, probably indicating that the intervention could not prevent the ongoing damage to the immature white matter resulting from the presence of blood, increased intraventricular pressure, or both.

Whether the timing of the intervention makes a real difference in outcomes requires further study. There is scarce information on the effect of PHVD on the progression of brain injury and brain growth in the developing brain. In a case-control study, Brouwer et al. [13] evaluated the impact of GMH-IVH and PHVD on brain volumes on TEA-MRI and found that PHVD was independently associated with lower deep gray matter and cerebellar volumes and increased ventricular volumes. In Brouwer’s study, white matter volume was not reduced, although the authors observed increased apparent diffusion coefficients in the posterior white matter suggestive of abnormalities in white matter integrity. Our intervention protocol is based on a high-threshold approach (VI >p97+4 mm and AHW >10 mm) (according to de Vries et al. [23]) unlike Brouwer’s study where the majority of infants with PHVD received an earlier intervention, starting before the ventricular index crossed the p97+4 mm threshold according to Levene’s nomogram [29]. Other studies have reported better outcomes with earlier intervention in PHVD [34, 43]. Cizmeci et al. [34], in a nested population of the Early vs. Late Ventricular Intervention Study (ELVIS) that used a structured MRI global brain abnormality score to assess brain injury, showed that infants in the low-threshold group (VI >p97 and AHW >6 mm) had better outcomes than those in the high-threshold group (VI >p97 + 4 mm and AHW >10 mm), with 46% of infants with normal/mild scores in the low-threshold group compared to 11% in high-threshold group. The authors also showed statistically significant differences between the groups in myelination delay, thinning of the corpus callosum, and dilatation of the lateral ventricles. In this study, VI and AHW on TEA-MRI revealed smaller ventricles in the low-threshold group compared with the high-threshold group (VI 13.4 vs. 15.9 mm and AHW 6.6 vs. 10.6 mm). Cizmeci’s findings on the high-threshold group are similar to those found in our study with an VI of 18.4 and an AHW of 10.9 mm. It therefore appears that the timing of the intervention has a real effect on structural brain injury. Damage to the immature white matter can occur as the consequence of a myriad of factors [16, 18–21, 44, 45]. The benefits of the low-threshold intervention strategy could be related to the earlier wash-out of blood products and free radicals, as well as intraventricular pressure release, potentially decreasing the risk of neuroinflammation [22, 23].

An early intervention in PHVD has also been advocated as a measure to reduce the need for VSI [5, 43]. In a retrospective study, de Vries et al. [5] observed that preterm infants with PHVD receiving a late approach strategy for cerebrospinal fluid removal (VI ≥p97 + 4 mm) required VSI more frequently and showed higher rates of neurodevelopmental impairment than infants undergoing an early approach (VI <p97 + 4 mm). However, the ELVIS trial [23] found no differences in the need for VSI (19% in the low-threshold and 23% in the high-threshold group). Our VSI rate was 30% of the cases diagnosed as PHVD, which is within the reported range (30–60%) [3, 5, 22, 46]. More studies are needed to determine whether an early intervention with prompt removal of blood products can reduce the need for VSI.

This study has several limitations such as its retrospective nature, the small number of included patients and the loss of data in white matter assessment in 15% of the cohort due to early death or poor quality cUS images. However, the strength of this study is that the study population represents the evolving nature of WMI associated with PHVD in a population of preterm infants in whom a standardized protocol based on high-threshold intervention was employed.

In summary, diffuse WMI eventually leading to white matter loss is a frequent finding in preterm infants with grade 2–3 GMH-IVH who develop PHVD. Consistent with the hypothesis of permanent WMI, many of the infants showed severe ventricular dilatation regardless of cerebrospinal fluid removal through a ventricular reservoir or VSI. Despite a systematic surveillance based on serial ventricular measurements and an intervention plan guided by cUS ventricular index, a significant proportion of the infants in our cohort presented significant ventricular enlargement at TEA, indicating either the co-occurrence of brain injury at the time of bleeding or ongoing damage related to inflammation or pressure. Strategies to prevent severe bleeding and implement earlier intervention protocols would ultimately address the pathophysiology of this process and might reduce the impact of GMH-IVH and PHVD on WMI.

## Supporting information

S1 File Study’s underlying data set. (SAV)
