# Correction: Posthemorrhagic ventricular dilatation late intervention threshold and associated brain injury

**DOI:** 10.1371/journal.pone.0315515

**Published:** 2024-12-05

**Authors:** Eva Valverde, Marta Ybarra, Andrea V. Benito, María Carmen Bravo, Adelina Pellicer

The Fig 2 is at low resolution. Please see the correct Fig 2 here.

**Fig 1:**
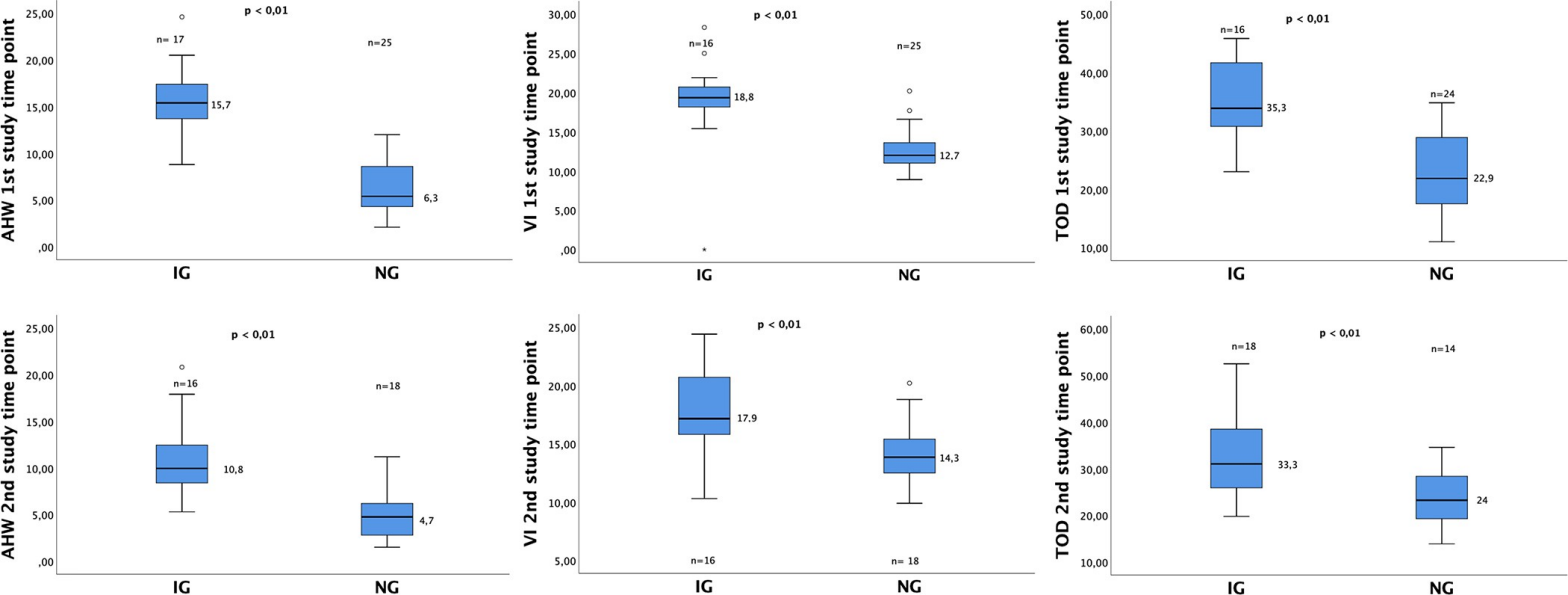


## References

[1] Valverde E, Ybarra M, Benito AV, Bravo MC, Pellicer A (2022) Posthemorrhagic ventricular dilatation late intervention threshold and associated brain injury. PLOS ONE 17(10): e0276446. 10.1371/journal.pone.0276446 36301835 PMC9612444

